# Exosome‐mediated effects and applications in inflammatory bowel disease

**DOI:** 10.1111/brv.12608

**Published:** 2020-05-14

**Authors:** Dickson K. W. Ocansey, Li Zhang, Yifei Wang, Yongmin Yan, Hui Qian, Xu Zhang, Wenrong Xu, Fei Mao

**Affiliations:** ^1^ Key Laboratory of Medical Science and Laboratory Medicine of Jiangsu Province, School of Medicine Jiangsu University 301 Xuefu Road Zhenjiang Jiangsu 212013 China; ^2^ Directorate of University Health Services, University of Cape Coast, PMB Cape Coast Ghana; ^3^ Nanjing Lishui People's Hospital, Zhongda Hospital Lishui Branch Southeast University Nanjing Jiangsu 211200 China

**Keywords:** exosome, inflammatory bowel disease, microenvironment, immune system cells, gut microbiota, intestinal mucosal barrier, therapy

## Abstract

Gut mucosal barriers, including chemical and physical barriers, spatially separate the gut microbiota from the host immune system to prevent unwanted immune responses that could lead to intestinal inflammation. In inflammatory bowel disease (IBD), there is mucosal barrier dysfunction coupled with immune dysregulation and dysbiosis. The discovery of exosomes as regulators of vital functions in both physiological and pathological processes has generated much research interest. Interestingly, exosomes not only serve as natural nanocarriers for the delivery of functional RNAs, proteins, and synthetic drugs or molecules, but also show potential for clinical applications in tissue repair and regeneration as well as disease diagnosis and prognosis. Biological or chemical modification of exosomes can broaden, change and enhance their therapeutic capability. We review the modulatory effects of exosomal proteins, RNAs and lipids on IBD components such as immune cells, the gut microbiota and the intestinal mucosal barrier. Mechanisms involved in regulating these factors towards attenuating IBD have been explored in several studies employing exosomes derived from different sources. We discuss the potential utility of exosomes as diagnostic markers and drug delivery systems, as well as the application of modified exosomes in IBD.

## I. INTRODUCTION

**Fig 1:**
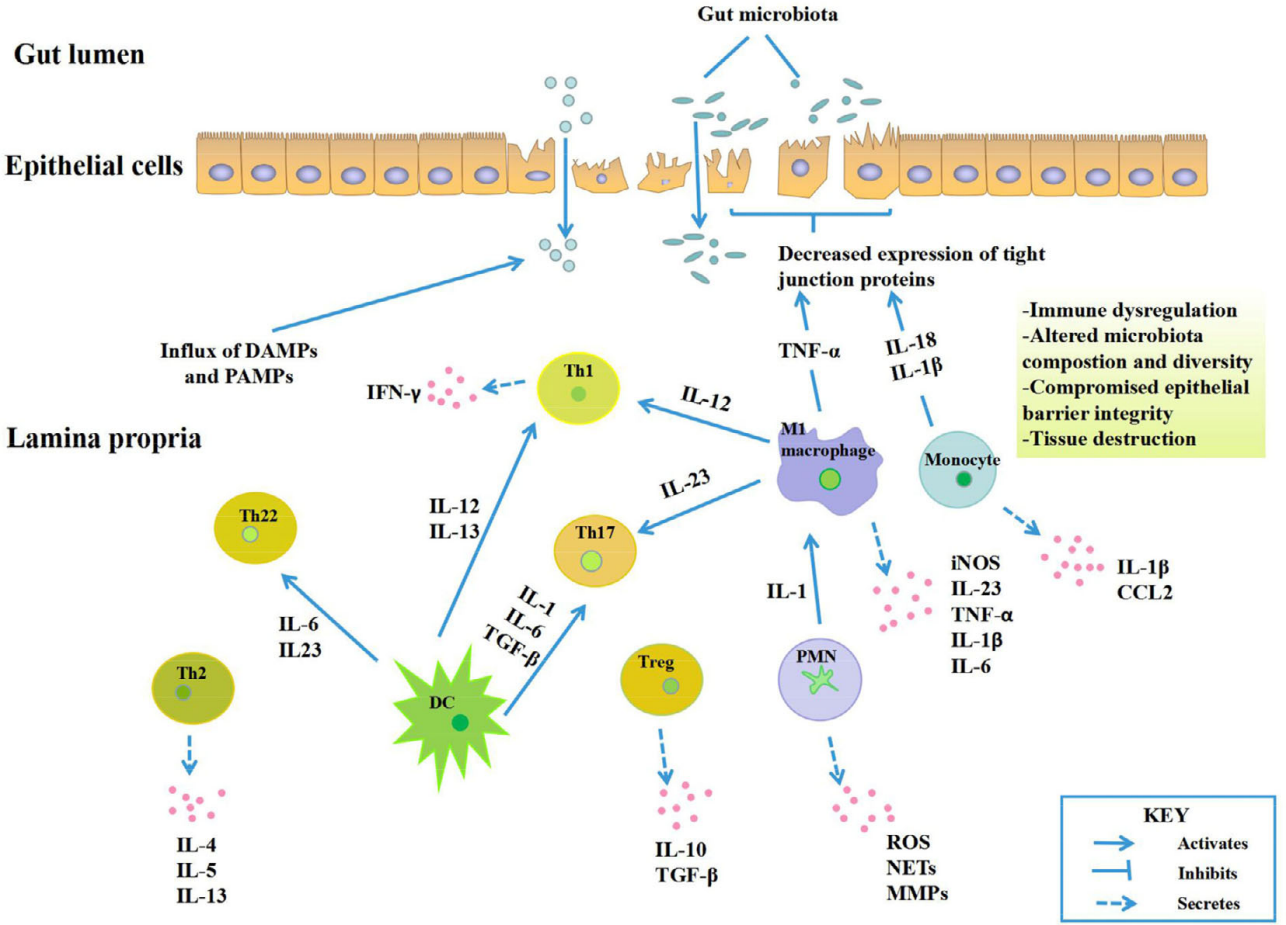
The active inflammatory bowel disease (IBD) microenvironment. Within the inflamed bowel, increased recruitment takes place of innate immune cells such as macrophages, monocytes, dendritic cells, neutrophils and T‐cells. The inflammatory cascade continues *via* cytokines secreted by these cells and other chemokines expressed in the IBD microenvironment. Together these elements lead to dysregulation, dysbiosis, and compromised intestinal barrier integrity. CCL2, chemokine c‐c motif ligand 2; DAMPs, damage‐associated molecular patterns; DC, dendritic cell; IFN‐γ, interferon gamma; IL, interleukin; iNOS, inducible nitric oxide synthase; MMPs, matrix metalloproteinases; NETs, neutrophil extracellular traps; PAMPs, pathogen‐associated molecular patterns; PMN, polymorphonuclear leukocytes, ROS, reactive oxygen species; TGF‐β, transforming growth factor β; Th, T helper; TNF‐α, tumour necrosis factor α; Treg, regulatory T cells.

Inflammatory bowel disease (IBD) is a term used to describe a group of chronic autoimmune diseases, including ulcerative colitis (UC) and Crohn's disease (CD), that affect the gastrointestinal tract. The characteristic prolonged inflammation coupled with immune dysregulation result in damage to the gastrointestinal tract. Although the pathogenesis of IBD still remains unclear, its mechanism is believed to be associated with genetic, environmental, gut microbiota and immune response factors (Ocansey *et al*., 2019). At the site of intestinal mucosal inflammation, profound microenvironmental changes take place including depletion of local nutrients, imbalances in tissue oxygen supply and demand and the production of high quantities of reactive nitrogen and oxygen intermediates. The resultant oxidative stress, endoplasmic reticulum (ER) stress, and hypoxia stress response signalling affect mucosal barrier integrity, cell survival, immunomodulation, as well as the composition, diversity and metabolic profiles of the gut microbiota (Taylor & Colgan, 2007; Cao, 2018). Within the IBD microenvironment (Fig. 1), there is a dynamic and complex interplay between immune and non‐immune cells *via* the mediation of secreted cytokines, which invariably participate in the perpetuation and amplification of the IBD‐associated inflammatory cascade (Marafini *et al*., 2019). Inflammatory cells such as polymorphonuclear neutrophils, macrophages and monocytes, and adaptive immune cells such as local T and B cells are recruited in response to chemokine signals produced within the site of active inflammation, such as interleukin 8 (IL‐8), leukotriene B4 (LTB4), platelet‐activating factor (PAF), complement factor C5a, and N‐formylated peptides (Colgan, Curtis, & Campbell, 2013; Campbell, Kao, & Colgan, 2016; Marafini *et al*., 2019).

IBD therapies seek to correct immune dysregulation and dampen inflammation within the intestinal mucosa. Amongst such therapies is exosome‐based therapy. As extracellular vesicles (EVs), exosomes are released by different types of cells and contain a variety of functional units mainly proteins, nucleic acids and lipids. Based on their endogenous properties and multifunctional abilities, these 30–150 nm lipid bilayer membrane vesicles have generated much recent interest in the search for medicines and pharmaceutical interventions for autoimmune diseases (including IBD) and several other conditions such as heart disease, cognitive decline, diabetes, and bone and muscle conditions (Phinney & Pittenger, 2017; Samanta *et al*., 2018; Yang *et al*., 2019). Exosomes play central roles in cell–cell communication that result in the modulation of several pathophysiological pathways such as immune responses in inflammation, infection and tumour growth, *via* vesicular transport and delivery of proteins and nucleic acids to recipient cells (Barile & Vassalli, 2017). Within the IBD microenvironment, exosomes modulate factors such as immune system cells, the gut microbiota, and the intestinal barrier as part of the mechanism to repair damage and restore intestinal mucosal functions. Herein, we review the functional effects of exosomal components in IBD attenuation, particularly the modulatory effects of exosomes on immune system cells, the gut microbiome, and intestinal barrier integrity in the treatment of IBD. We also discuss the application of exosomal components as potential biomarkers of IBD and the use of modified exosomes in IBD treatment.

## II. GENERAL FUNCTIONS AND COMPOSITION OF EXOSOMES

Exosomes are actively secreted from cells through an exocytosis pathway during crosstalk between cells and in receptor removal mechanisms. This pathway involves initiation of activated growth factor receptors located on the plasma membrane surface (Stoorvogel *et al*., 2002). These 30–150 nm membrane vesicles are generated in the endosomal compartment and function in intercellular modulation in both physiological and pathological activities. They play critical roles in the immune system by modulating immune responses due to their potent immune‐activating or immunosuppressive effects (Tan *et al*., 2016). Exosomes are also essential for neuronal cell communication that improves cellular functions such as the ability of microglia to support axonal integrity and myelination. It has been demonstrated that, under oxidative stress conditions, neurons survive better in the presence of oligodendrolglial exosomes, and that this process involves the Akt and extracellular‐regulated kinase (ERK) signalling pathways (Fröhlich *et al*., 2014). The cardiovascular system also relies on exosomes for normal function. Cardiomyocytes secrete exosomes constitutively, but the quantity released triples under conditions of hypoxia or cellular stress (Gupta & Knowlton, 2007). This increased secretion together with upregulated exosomal tumor necrosis factor‐α (TNF‐α) expression induces cardiomyocyte apoptosis (Yu *et al*., 2012).

The functional effects of exosomes have been well studied in many pathological and non‐pathological conditions including diabetic cardiomyopathy (Salem & Fan, 2017), cardiovascular disease and cardioprotection (Barile *et al*., 2017; Suzuki *et al*., 2017), joint diseases (Li *et al*., 2018*b*), wound healing (Golchin, Hosseinzadeh, & Ardeshirylajimi, 2018), Alzheimer's Disease (Malm, Loppi, & Kanninen, 2016), placental homeostasis and pregnancy disorders (Salomon & Rice, 2017), viral disease (Anderson, Kashanchi, & Jacobson, 2016), and various cancers (Kibria *et al*., 2018; Sundararajan, Sarkar, & Ramasamy, 2018) among others. Although mechanisms involved in the biogenesis and secretion of exosomes are still not fully understood, certain vital factors or mechanisms that control the composition and hence the functions and secretion of exosomes have been documented (Palmulli & van Niel, 2018).

Exosomes consist of varying compositions of macromolecules mainly proteins, microRNAs (miRNAs), messenger RNAs (mRNAs) and lipids. Studies on exosomes from different sources indicate that they share common exosomal constituents. There are currently 41,860 exosomal protein, >7540 exosomal RNA, and 1116 exosomal lipid molecules documented from more than 286 exosome‐based studies annotated using the International Society for Extracellular Vesicles minimal experimental requirements for definition of EVs (Keerthikumar *et al*., 2016). To date, some of the common and cell‐type‐specific exosomal proteins documented include the Ras superfamily of monomeric G proteins (Rab), adhesion molecules, heat shock proteins (such as HSC73, HSC90), annexins I, II, V and VI, cytoskeletal proteins (synenin, actin, moesin, albumin), tetraspanins (CD9, CD63, CD81, CD82), and GTPases (Ferguson & Nguyen, 2016; Samanta *et al*., 2018). However, a recent study that reassessed exosome composition reported that cytoskeletal proteins, glycolytic enzymes, and argonaute 1–4 are not detected in exosomes. They further demonstrated that small EVs are not vehicles of active DNA release, but rather, extracellular DNAs are secreted *via* an autophagy and multivesicular‐endosome‐dependent but exosome‐independent mechanism (Jeppesen *et al*., 2019).

Lipids found in exosomal compositional studies include cholesterol, phospholipids (e.g. phosphatidic acid, phosphatidylserine), diglycerides, glycerophospholipids, arachidonic acid (polyunsaturated omega‐6 fatty acid), and sphingolipids like ceramide and sphingomyelin. Compared to the parental cell, lipids are found in exosomes at ratios up to fourfold higher, which could possibly explain the increased membrane rigidity of exosomes. Furthermore, specific bioactive lipids are found in exosomes, particularly leukotrienes, prostaglandin, and activated enzymes of lipid metabolism (Subra *et al*., 2010; Skotland, Sandvig, & Llorente, 2017; Skotland *et al*., 2019; Zhang *et al*., 2019).

**Fig 2:**
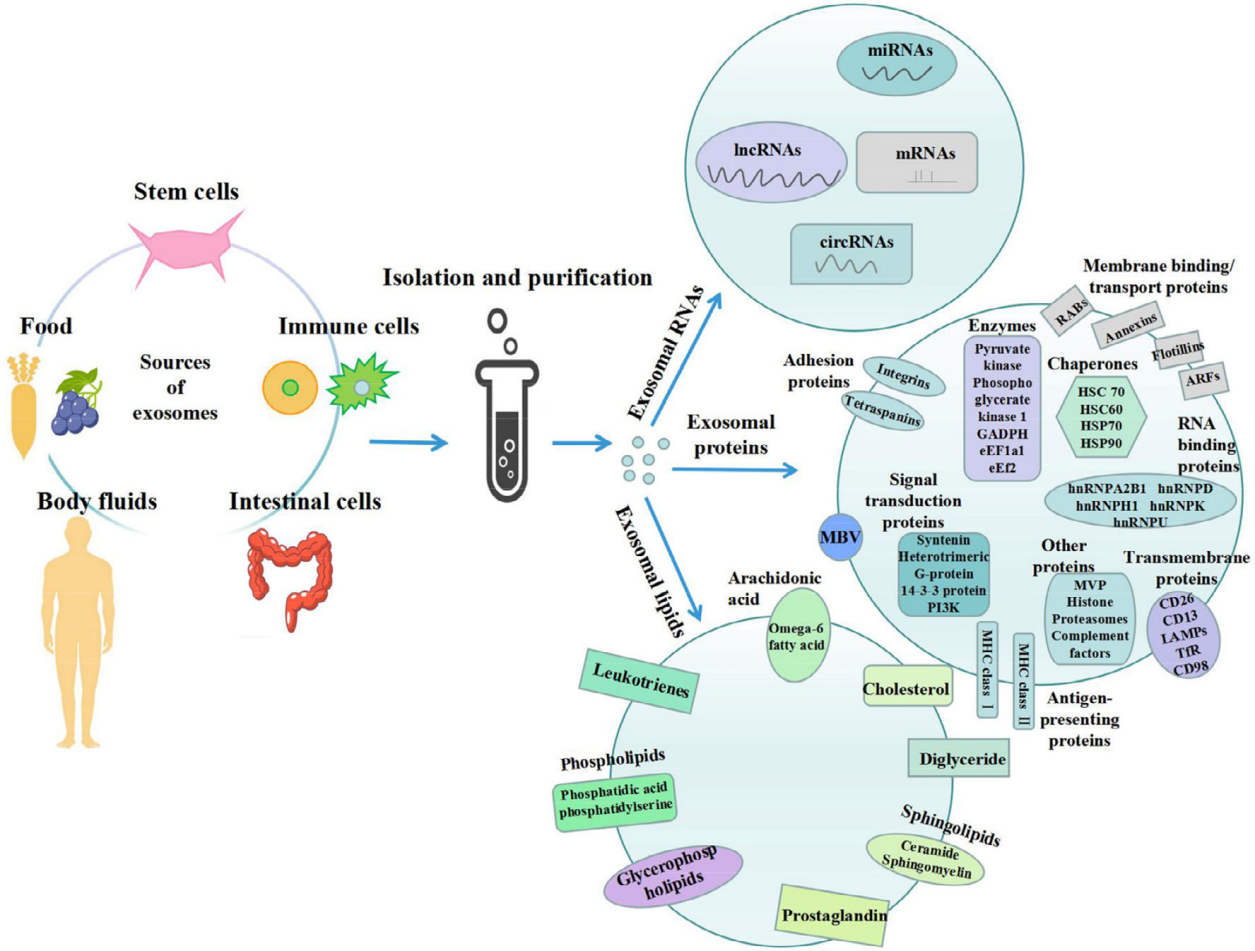
Sources and composition of exosomes. Regardless of their source, exosomes share a similar structure and composition. The variety of molecules in exosomes are functionally characterized and grouped into exosomal RNAs, proteins and lipids. These cargos and structural components are further separated into functional units within these broad groups. ARF, ADP‐ribosylation factor; CD, cluster of differentiation; circRNA, circular RNA; eEF, eukaryotic translation elongation factor; GAPDH, glyceraldehyde‐3‐phosphate dehydrogenase; HNRNP, heterogeneous nuclear ribonucleoprotein; HSC, heat shock cognate, HSP, heat shock protein; LAMP, limbic system‐associated membrane protein; lncRNA, long non‐coding RNA; MBV, matrix‐bound nanovesicles; MCH, major histocompatibility complex; miRNA, microRNA; mRNA, messenger RNA; MVP, major vault protein; PI3K, phosphoinositide 3‐kinase; RAB, Rab protein; TFR, transferrin receptor.

In addition to these proteins and lipids, several exosomal RNAs have also been reported. These RNAs encapsulated in the exosome mainly participate in cell cycle progression, cellular migration, angiogenesis, or histone modification. Exosomal miRNA takes part in modulating gene expression in relation to stem cell differentiation, haematopoiesis, organogenesis, tumorigenesis and tumour metastasis (Zhang *et al*., 2015*a*; Isola & Chen, 2016; Kalluri, 2016). An exosomal RNA database reports 18,333 mRNAs, 58,330 circular RNAs (circRNAs), and 15,501 long non‐coding RNAs (lncRNAs) (Li *et al*., 2018*a*). Studies have been carried out on the role of these exosomal long RNA species (mRNAs, circRNAs, and lncRNAs) in tumour formation and progression with a focus on their use as potential targets in the diagnosis and treatment of tumours (Zhou *et al*., 2018*b*). Statello *et al*. (2018) report that certain RNA binding proteins (RBPs) serve as key players in transporting RNAs into exosomes. They demonstrate the presence of 30 RBPs in exosomes that interact with RNAs to form RNA–RBP complexes with both exosomal RNAs and cellular RNAs. The RNA–RBP complexes were found within the exosomes in the form of RNA–ribonucleoprotein complexes (Statello *et al*., 2018). Figure 2 summarizes the available information on the sources and composition of exosomes.

## III. SOURCES OF EXOSOMES

Selection of the source of exosomes for therapy is of great importance because the constituents of the exosome, including its surface proteins and lipids, play vital roles in their functions. The surface markers on the source cells should also be studied to understand their biological characteristics since they could jeopardize the expected outcome and negatively impact the recipient.

### (1) Stem cells

Stem cell‐derived exosomes contribute to the self‐renewal, immunomodulatory, expansion and damage repair abilities of stem cells. Thus, stem cell‐derived exosomes have demonstrated significant therapeutic benefits in several diseases. Mao *et al*. (2017*a*) showed that mesenchymal stem cell (MSC)‐derived exosomes could relieve the symptoms of IBD and helped in the recovery of damaged tissues and organs.

### (2) Food

Recently, exosomes from food such as fruits have received interest based on the fact that these exosomes are frequently ingested, and thus are generally considered safe. These edible plant‐derived exosome‐like nanoparticles are known to possess anti‐inflammatory properties and contain microRNAs that are crucial in the mediation of pathological and physiological activities in both animals and plants. Xiao *et al*. (2018) identified several such nanovesicles from 11 edible vegetables and fruits. Upon analysis, it was revealed that they had a close association with the inflammatory response and cancer‐related pathways and were capable of regulating human mRNA (Xiao *et al*., 2018). Exosome‐like nanoparticles were isolated in another study from coconut water and other food items, with diameters smaller than those of animal‐derived exosomes (Zhao *et al*., 2018). Some of these food‐derived vesicles have been applied in experimental animal models with encouraging outcomes, although no clinical trials have been published to date.

### (3) Immune cells

Exosomes derived from immune cells have also attracted intense research interest. They have been applied in vaccine and drug‐delivery studies and have produced significant therapeutic outcomes (Cheng, Wang, & Huang, 2017; Jiang & Gao, 2017). Macrophages, monocytes and dendritic cells (DCs) are key immune cells from which exosomes have been derived for therapeutic studies. Immune cell‐derived exosomes have been proved to evade the immune system and escape clearance, thereby extending their circulation period and therapeutic impact.

### (4) Body fluids

Exosomes have been isolated and studied from blood (Baranyai *et al*., 2015), amniotic fluid (Xiao *et al*., 2016), urine (Street *et al*., 2017), saliva (Liu *et al*., 2018*b*), and breast milk (Hock *et al*., 2017). In one of these studies, Hock *et al*. (2017) reported that exosomes extracted from rat breast milk significantly increased intestinal epithelial cell (IEC) viability, proliferation and stem cell activity. By contrast, other studies have associated breast milk‐derived exosomes with the promotion of epithelial mesenchymal transition (EMT) (Qin *et al*., 2016).

### (5) IECs

IECs provide a physical and biochemical barrier that separates the commensal microbiome from host tissue to maintain intestinal homeostasis. Secretory IECs assist this function *via* the secretion of antimicrobial peptides and mucins. Exosomes derived from these cells have been shown to play important roles in IEC‐induced immune tolerance, and to function critically in exosome‐mediated immune responses in the pathogenesis of IBD (Xu *et al*., 2016). Exosomes secreted by IECs bear exogenous peptides complexed to major histocompatibility complex class II (MHC‐II) and preferentially interact with DCs, resulting in greatly enhanced peptide presentation to T cells. These exosomes have been shown to link local immune cells and luminal antigens powerfully through the mediated transfer of small quantities of luminal antigenic information, and to facilitate immune surveillance at mucosal surfaces (Mallegol *et al*., 2007).

### (6) Tumours

Exosomes present novel targets in cancer medicine with both therapeutic and diagnostic applications. Due to their role in cell‐to‐cell communication, these vesicles affect both tumour progression and metastasis (Sundararajan *et al*., 2018). In addition to being cancer biomarkers, exosomes could be engineered to reinstate tumour immunity. Tumour‐derived exosomes interact with a variety of cells within the tumour microenvironment to create tumour‐friendly changes which promote stromal activation, increased vascular permeability, induction of angiogenic switch, and immune escape of tumour cells (Brinton *et al*., 2015). They further aid the establishment of the pre‐metastatic niche, promote EMT, transmit chemo‐resistance abilities to nearby cells, and defend tumour cells against the cytotoxic effects of drugs and immune cells (Brinton *et al*., 2015; Zhang *et al*., 2015*b*). There is much potential for the discovery of exosome‐based diagnostic and therapeutic tools in the treatment of cancer.

## IV. EXOSOME‐INDUCED MODULATION IN IBD

Exosomes contain molecular particles from their parental cells, including RNAs and proteins. While the composition of exosomal proteins differs according to the cell and tissue of origin, they also have an evolutionarily conserved common set of protein molecules. These molecular constituents are responsible for the functional activities of the exosome. Exosomes are involved in immunomodulation *via* the functional transfer of miRNAs, mRNAs and other constituents between immune cells. Xu *et al*. (2016) studied two small GTPases involved in exosomal secretion (RAB27A and RAB27B) and reported increased levels of RAB27A‐ and RAB27B‐positive immune cells in the colonic mucosa of individuals with active UC in comparison with healthy controls (Xu *et al*., 2016). This indicates the crucial role of exosome‐mediated immune responses in the pathogenesis of IBD. Further investigations of exosomal components with respect to IBD pathogenesis, progression, diagnosis and treatment are likely to lead to important advances.

### (1) Exosomal proteins

Typical exosomal‐associated proteins linked with the regulatory functions of the exosome include the endosomal proteins Alix and tumour susceptibility gene 101 (TSG101), and the tetraspanin proteins CD63, CD9 and CD81. The therapeutic potential of exosomes is usually rationalized on the basis of the presence of biologically relevant proteins or RNAs in exosomes. However, a recent review (Toh *et al*., 2018) based on MSC‐derived exosomes, considered their physical presence, biochemical functionalities, biologically relevant concentrations, as well as their potential to elicit appropriate timely biochemical responses. They conclude that MSC‐derived exosomes most probably function *via* protein‐ rather than RNA‐based mechanisms (Toh *et al*., 2018).

#### (a) *Immune cell modulation*

Macrophages are important in the maintenance of intestinal homeostasis, and their regulation is reported to be crucial in the pathogenesis of IBD, with some evidence to support the involvement of exosomes in the activation of macrophages. Systemically administered exosomes obtained from bone marrow MSCs greatly attenuate colitis in various models of IBD (Liu *et al*., 2019). The treatment caused polarization of M2b macrophages without leading to intestinal fibrosis, maintained the integrity of the intestinal barrier and down‐modulated inflammatory responses. These exosomes were found to be enriched in proteins that participate in the regulation of multiple biological anti‐colitis activities, particularly metallothionein‐2 which is involved in the inhibition of inflammatory responses (Liu *et al*., 2019). Wong *et al*. (2016) investigated the potential participation of circulating exosomal proteins in the activation of macrophages by inducing acute colitis in mice and isolating their serum exosomes. Proteomic and bioinformatics analysis of the serum after treatment with RAW264.7 macrophages identified 56 proteins (mainly acute‐phase proteins and immunoglobulins) which are particularly involved in the complement and coagulation cascade, leading to activation of macrophages (Wong *et al*., 2016). This finding links the modulation of macrophages by exosomal proteins with IBD pathogenesis.

**Fig 3:**
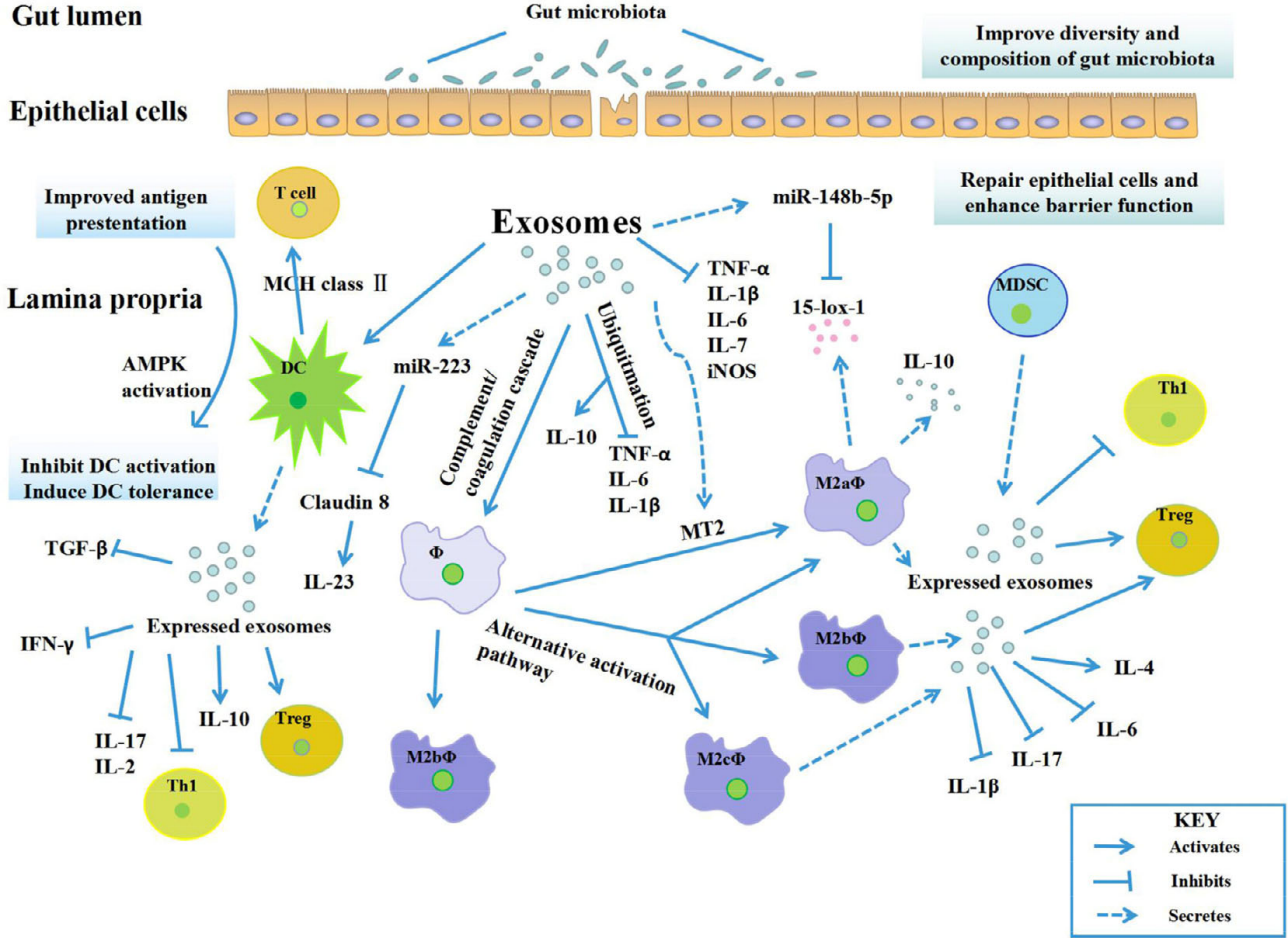
Exosomal‐induced modulation of immune system cells. The administration of exosomes into the inflammatory bowel disease (IBD) environment promotes anti‐inflammatory responses *via* polarizing macrophages into the M2 phenotype, inhibiting dendritic cell activation and inducing their immune tolerance, and triggering regulatory T cells (Treg) activation while inhibiting T helper type 1 (Th1) cells. Exosome‐treated immune cells further express exosomes that encourage anti‐inflammatory responses. In summary, the functional exosomal components that are expressed inhibit inflammatory and pro‐inflammatory factors, and promote anti‐inflammatory factors. AMPK, AMP‐activated protein kinase; DC, dendritic cell; IFN‐γ, interferon gamma; IL, interleukin; iNOS, inducible nitric oxide synthase; M, macrophage; MCH, major histocompatibility complex; MDSC, myeloid‐derived suppressor cell; miR, microRNA; MT2, melanotan 2; TGF‐β, transforming growth factor β; Th, T helper; TNF‐α, tumour necrosis factor α; Treg, regulatory T cells; 15‐lox‐1,15‐lipoxygenase‐1; φ, macrophage.

Hookworms are known to create an immunomodulatory environment within their host. Their ability to suppress inflammation effectively led to their use in clinical trials to treat IBD (Helmby, 2015). Recently, helminth‐secreted exosome‐like EVs have been characterized and their functions in host–parasite interactions investigated (Eichenberger *et al*., 2018*b*). EVs from *Nippostrongylus brasiliensis* (a roundworm used as a model for human hookworm) contained 81 proteins including common exosomal proteins such as tetraspanin, 14‐3‐3 protein, enolase and heat shock proteins, together with 52 miRNA species. These components acted to protect mice against colitis inflammation by significantly suppressing cytokines [γ‐interferon (IFNγ), IL‐6,IL‐1β, and IL‐17a] related to colitis pathology and upregulating anti‐inflammatory cytokine IL‐10 (Eichenberger *et al*., 2018*a*). Figure 3 shows the general modulatory features of exosomes on the immune system.

#### (b) *Gut‐microbiome modulation*

Many factors including genetic, microbial, dietary, environmental and chemical can lead to altered gut‐microbiome composition and diversity, causing dysbiosis – a characteristic marker of IBD (Ocansey *et al*., 2019). The ability of exosomes to regulate the gut microbiome needs further exploration. The functions of several exosomal heat shock proteins such as HSP20, HSP27, HSP70, HSP72, HSP73, αBC and HSP90 have been investigated, including in the pathogenesis of IBD (Clayton, 2005; Samborski & Grzymisławski, 2015; Reddy *et al*., 2018). Examples of such modulatory influences on the microbiome are exosomal HSP70 utilization of gram‐negative bacteria receptors (Toll‐like receptor 4; TLR4) and gram‐positive bacteria receptors (TLR2) to stimulate proinflammatory responses, and exosomal HSP72 in the functions of IECs (Asea *et al*., 2002; Samborski & Grzymisławski, 2015).

Breast milk has been shown to contain a diverse array of microbiota together with many biologically active components like exosomes that assist in mucosal tissue, immune system and microbiome development and maintenance (Le Doare *et al*., 2018). van Herwijnen *et al*. (2016) analysed protein constituents of milk‐derived EVs and reported a total of 1963 proteins, including common EV‐associated proteins such as CD9, Flotillin‐1, and Annexin A5, as well as 633 unidentified proteins. Such active EV components are likely to participate in gut‐microbiome modulation in both the normal and IBD mucosal microenvironment: bovine milk‐derived exosomes alter gut microbiome diversity and composition in mice (Zhou *et al*., 2019). This further emphasizes that exosomes and their cargos do not merely regulate gut‐microbiome communities but actively participate in crosstalk between bacteria and their hosts.

#### (c) *Intestinal barrier modulation*

IECs are an indispensable component of the intestinal barrier. Studies have shown that IEC‐6 cells can take up exosomes derived from milk, which in turn regulate the functions of the intestinal barrier. Exosomal proteins such as TSG101, CD63, and HSP70 are found in high concentrations in yak milk‐derived exosomes that facilitate intestinal cell survival and proliferation (Gao *et al*., 2019). Further proteomic analysis of 9430 unique milk colostrum‐derived exosomal proteins revealed that the exosomes were highly enriched in proteins associated with cellular growth and immune modulation (Samuel *et al*., 2017). In several other studies, exosomes derived from different types of milk have been documented to enhance IEC viability, proliferation, stem cell activities and the development of intestinal tract (Chen *et al*., 2016; Hock *et al*., 2017). Exosomes from bone‐marrow MSCs significantly restore abnormal intestinal barrier functions by decreasing intestinal permeability and reducing infiltration of gut microbiota into the lamina propria. The mRNA levels of colonic antimicrobial proteins such as lysozyme 1 (Lyz1), angiogenin‐4 and the defensins Defa29 and Defa20 were also restored. The exosomal protein metallothionein‐2 was particularly necessary for the anti‐inflammatory responses (Liu *et al*., 2019).

### (2) Exosomal nucleic acids

RNAs are relevant in inflammatory processes, including their established involvement in the modulation of the intestinal immune system, barrier functions and microbiota, as well as having a role in the pathogenesis of IBD.

#### (a) *Immune cell modulation*

IECs form a single‐layered lining to the intestines, linked to neighbouring cells through tight junctions and anchored to the extracellular matrix. They may use cargos of exosomes to communicate molecular or genetic information to distant cells or to the gut microbiome. Exosomal secretion from IECs occurs at the basolateral and apical sides. Whereas basolateral exosome secretion elicits local immune responses by presenting luminal antigens to lymphocytes, apical secretion enhances the secretion of anti‐microbial peptides (Smythies & Smythies, 2014). Hu *et al*. (2013) reported that luminal secretion of IEC exosomes occurs after pathogenic infection has downregulated the miRNA let‐7, leading to increased delivery of anti‐microbial peptides to inhibit the pathogen. This reveals the presence of a miRNA‐associated regulatory loop between IECs and pathogens (Hu *et al*., 2013). Furthermore, basolateral release of IEC exosomes activates DCs to present exogenous peptides (*via* MHC class II) to T‐cells, although little is known about miRNA involvement in this mechanism (Mallegol *et al*., 2007). The role of lncRNAs in IBD has been documented in many studies. These RNAs are enriched in exosomes with their expression levels in exosomes dependent on the cell of origin (Dragomir, Chen, & Calin, 2018). Liu *et al*. (2018*a*) report that inhibition of the lncRNA NEAT1 (nuclear enriched abundant transcript 1) suppresses the inflammatory response in IBD *via* regulating exosome‐mediated polarization of macrophages and the intestinal epithelial barrier. miR‐21 (enriched in exosomes) has been implicated in different phases of IBD pathogenesis. The deletion of miR‐21 attenuated the symptoms associated with a T‐cell transfer colitis model, and a 2,4,6‐trinitrobenzenesulfonic acid (TNBS)‐induced colitis model (Wu *et al*., 2014). On the other hand, levels of miR‐21 were reduced in T cells obtained from colonic mucosa during the recovery stage of UC (Ando *et al*., 2016). These data suggest that miR‐21 both appears to execute its versatile functions in a colitis‐dependent manner, and also seems involved in the modulation of T‐cell functions to regulate IBD.

Another nucleic acid highly enriched in exosomes including MSC‐derived exosomes is miR‐223 (Wang *et al*., 2015*b*). Existing data on the role of miR‐223 in IBD is seemingly contradictory. On the one hand, miR‐223 suppresses gene expression of claudin‐8, leading to activation of the IL‐23 pathway, which is critical for IBD progression (Wang *et al*., 2016*a*). On the other hand, however, mice deficient in miR‐223 were found to be more susceptible to induced colitis, suggesting a modulatory role of miR‐223 in the maintenance of intestinal homeostasis. These mice exhibited reduced levels of intestinal CX3CR1 (hi) macrophages, accompanied by high levels of expression of a pro‐inflammatory phenotype in intestinal DCs and macrophages (Zhou *et al*., 2015). Moreover, human umbilical cord‐derived MSCs transfected with mimics of miR148b‐5p showed increased expression of miR148b‐5p in MSCs, leading to downregulation of the expression of 15‐lox‐1 in macrophages to promote colonic tissue repair in IBD (Kang *et al*., 2019).

#### (b) *Gut‐microbiome modulation*

**Fig 4:**
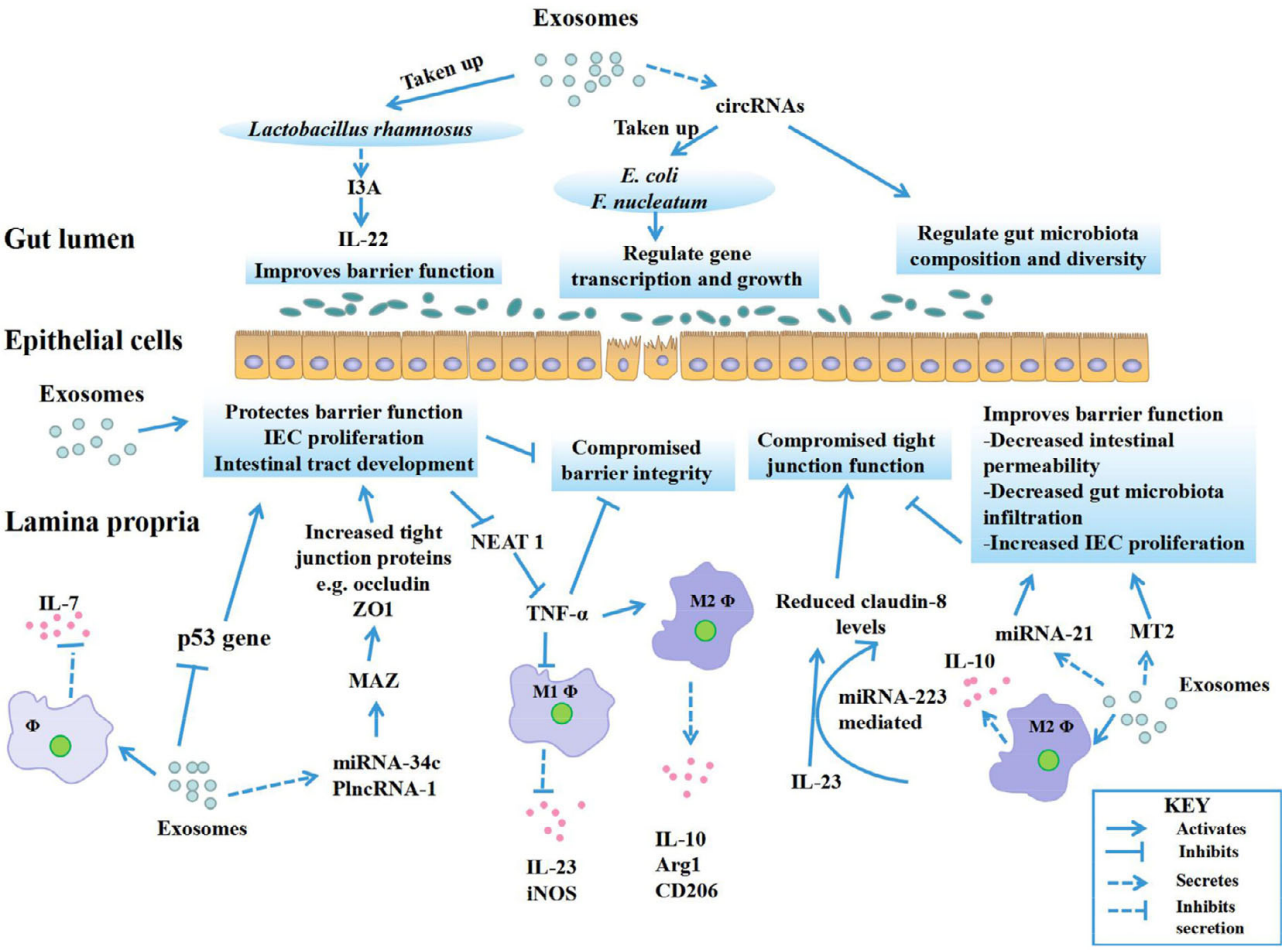
Exosomal‐induced modulation of the gut microbiota and intestinal barrier in inflammatory bowel disease (IBD). Exosomes, through their functional components, directly or indirectly interact with gut microbiota and intestinal epithelial cells (IECs). Some gut microbes take up exosome components which in turn regulate their gene transcription and growth, producing improved microbiota diversity and composition, and restoring barrier integrity. Exosomes also directly induce the proliferation of IECs and the development of the intestinal tract, and indirectly enhance barrier functions by inhibiting components of the inflammatory environment that negatively impact tight junction molecules and IECs. Arg1, Arginase 1; CD206, cluster of differentiation 206; circRNA, circular RNA; I3A, ingenol‐3‐mebutate; IEC, intestinal epithelial cell; IL, interleukin; iNOS, inducible nitric oxide synthase; M, macrophage; MAZ, myc‐associated zinc finger protein; miRNA, microRNA; MT2, melanotan 2; NEAT1, nuclear paraspeckle assembly transcript 1; PlncRNA1, long non‐coding RNA PlncRNA‐1; TNF‐α, tumour necrosis factor α; ZO‐1, zona occludens‐1; φ, macrophage.

The gut microbiome can be altered by dietary interventions to treat and prevent several diseases (Sonnenburg *et al*., 2016). In an experiment investigating mechanisms by which food substances modulate mucosal commensals, Teng *et al*. (2018) demonstrate that plant‐derived exosome‐like nanoparticles (ELNs) containing certain RNAs are taken up by the gut microbiome resulting in altered microbiome composition as well as host physiology. They further report that ginger‐derived ELNs (containing microRNA mdo‐miR7267‐3p) are preferentially taken up by Lactobacillaceae leading to targeting of several genes in *Lactobacillus rhamnosus*. The complex gene targeting mechanism yields increased expression of indole‐3‐carboxaldehyde (I3A), which subsequently induces IL‐22 production. This outcome is associated with intestinal barrier function improvement as well as amelioration of colitis in mice through an IL‐22‐dependent mechanism (Teng *et al*., 2018). Gut epithelial cells and homeodomain only protein X‐positive (Hopx^+^) cells are the main sources of faecal circulating miRNA. These miRNAs have been shown to enter gut‐bacteria like *Escherichia coli* and *Fusobacterium nucleatum* to control their gene transcription and growth. Transplanting these miRNAs restored faecal microbiota composition and also ameliorated induced colitis (Liu *et al*., 2016). Figure 4 summarizes exosomal effects on the gut microbiome and intestinal barrier functions.

#### (c) *Intestinal barrier modulation*

The general significance of miRNAs in IECs has been investigated. Dicer knockout in IECs of mice led to decreased goblet cell numbers, increased cryptic apoptosis, and inhibited intestinal barrier function, implying an important role of IEC miRNAs in supporting epithelial constitution and protection (McKenna *et al*., 2010). Functionally, IEC miRNAs contribute to the morphology and function of M cells, a crucial component of the mucosal antigen‐sampling system (Nakato *et al*., 2016). Chen *et al*. (2016) Detected six miRNAs that were significantly upregulated in IPEC‐J2 cells alongside other molecules like proliferating cell nuclear antigen (PCNA), caudal type homeobox 2 (CDX2), and insulin‐like growth factor 1 receptor (IGF‐1R), after treatment with milk‐derived exosomes. This upregulation coupled with inhibited expression of the p53 gene, promoted intestinal cell proliferation as well as intestinal tract development (Chen *et al*., 2016). Inhibition of the lncRNA NEAT1 suppresses the inflammatory response in IBD *via* modulation of the intestinal epithelial barrier and exosome‐mediated polarization of macrophages. NEAT1 Inhibition downregulates TNF‐α leading to increased activation of M2 macrophages and increased expression of the markers CD206, IL‐10, and arginase‐1(Arg‐1) (Liu *et al*., 2018*a*).

The crucial role of this network of lncRNA and miRNA has been analysed in diverse human diseases including IBD. One typical investigation documents that long non‐coding RNA PlncRNA‐1 and microRNA‐34c (miR‐34c) modulate the functions of the intestinal epithelial barrier through the regulation of tight junction proteins in IBD. The tight junction proteins zonula occludens‐1(ZO‐1) and occludin were modulated by myc‐associated zinc finger protein (MAZ). The expression of MAZ was in turn targeted by miR‐34c. Furthermore, upregulation of PlncRNA1 seemingly protected the intestinal epithelial barrier from dextran sulfate sodium (DSS)‐induced injury, while miR‐34c and PlncRNA1 coupled together to modulate ZO‐1, MAZ and occludin expression (Chen *et al*., 2017). Claudin‐8, a multigene family protein which forms the backbone of intestinal tight junctions, was discovered as a novel target of IL‐23 in IBD. Levels of claudin‐8 are appreciably decreased in IBD patients with inflamed colonic mucosa, and in TNBS‐induced colitis in mice (Wang *et al*., 2016*a*). miR‐223 (upregulated in IBD) is identified as a modulator of communication between the IL‐23 signal pathway and claudin‐8 in IBD development, hence acting as a modulator of the intestinal barrier (Wang *et al*., 2016*a*). Strategies to interrupt this crosstalk could represent novel therapeutic approaches for the management of IBD. Substance P (SP), a hormone or neuropeptide, and its receptor neurokinin‐1 receptor (NK‐1R) have been shown to modulate exosome biogenesis and exosome miRNA cargo sorting. The increased exosome production in turn promotes the migration and proliferation of target cells such as mouse colonic crypts and human colonic epithelial cells *via* exosomal miR‐21(Bakirtzi *et al*., 2019). Additionally, let‐7 miRNA and miR‐375 enhance Paneth cell differentiation and proliferation of intestinal epithelial stem cells (IESCs), respectively, in response to intestinal microbiota status (Park, Shimaoka, & Kiyono, 2017; Peck *et al*., 2017).

### (3) Exosomal lipids

Lipids are indispensable constituents of the exosome membrane, and it is known that specific lipids are enriched in exosomes relative to their parental cells. Recent investigations have focussed on the functions and composition of exosomal lipids, with over 1,116 lipid molecules from exosomes recorded in one database (Keerthikumar *et al*., 2016). Lipid composition analyses have been performed with exosomes derived from mast cells, DCs, reticulocytes, B cells, and basophilic leukaemia cells (Mathivanan *et al*., 2012). Notwithstanding, the roles of exosomal lipids and their possible use in therapeutics have not received sufficient attention. Lipids play important roles in exosome biogenesis and are characteristic of the cell type from which the exosomes originate: internal membranes of multivesicular bodies (MVBs) are enriched with lipids such as lyosbisphosphatidic acid (LBPA) which has important functions in exosome biogenesis, especially in intraluminal vesicle (ILV) formation (Chu, Witte, & Qi, 2005; Mathivanan *et al*., 2012). Exosome‐like nanoparticles obtained from grapes (GELNs) effectively protected mice from induced colitis, with further analysis indicating that the lipid components of the exosome‐like nanovesicles were crucial in inducing Lgr5^+^ stem cells, and the liposome‐like nanoparticles (LLNs) assembled with lipids from GELNs were required for *in vivo* targeting of intestinal stem cells (Ju *et al*., 2013).

Tight junction adhesion complexes are known to regulate epithelial barrier integrity, and some of these complexes reside in cholesterol‐enriched zones (known as lipid rafts) within cell membranes. It is reported that lipid rafts are disrupted in the ileum of mice with subclinical colitis, in IFN‐γ‐treated cells, and in UC patients with quiescent mucosal inflammation (Bowie *et al*., 2012). Thus these lipid rafts are distorted in the early stages of inflammation, leading to subsequent loss of mucosal barrier integrity. Lipid components of exosomes may help to reinstate lipid raft functions, hence restoring barrier integrity. They can also regulate immune system cells and restore the gut microbiome: cholesterol regulates experimental IBD to significantly reduce colonic histological scores and damage, and colonic inflammation (Xiong *et al*., 2016). n‐3 polyunsaturated fatty acids attenuate induced colitis by reducing inducible nitric oxide synthase (iNOS), cyclooxygenase‐2 (COX‐2), IL‐6, and LTB4 as well as regulating tight junction proteins (occludin, claudin‐1) and colonic mRNA levels of trefoil factor 3 (TFF3) and mucin 2 (MUC2) (Charpentier *et al*., 2018).

The lipid composition of exosomes and stability in their membrane structures give them significant advantages in applications as nanovehicles for intravenous injection. Exosomes expressed from cells that have been treated with an ether lipid precursor (hexadecylglycerol) demonstrate changes in both their lipid and protein composition (Phuyal *et al*., 2015). It is known that ether lipids are involved in membrane fusion, implying that increased fusion of MVBs with the plasma membrane could be achieved by the use of ether lipid precursors. Increased membrane fusion causes fewer intraluminal vesicles to be released, and the inhibition of enzymes and pathways associated with membrane fusion results in increased exosomal release (Skotland *et al*., 2017). These features could be utilized in the therapeutic application of exosomes. There remains, however, a need for sufficient studies into the therapeutic effects of exosomal lipids. The experimental studies available are summarized in Table 1.

**Table 1:** Exosome components and their effects in studies of inflammatory bowel disease (IBD)

Exosome component	Source	Effects	References
Metallothionein‐2	Bone marrow‐derived MSCs	Maintenance of intestinal barrier integrity Polarization of M2b macrophages Induction of IL‐10 from macrophages	Liu *et al*. (2019)
Annexin A1	IECs	Activates mucosal wound repair Potential biomarker of intestinal mucosal inflammation	Leoni *et al*. (2015)
81 proteins including tetraspanin 14–3‐3 protein, enolase and heat shock proteins, together with 52 miRNA species	Hookworm	Protection against colitis by significantly suppressing IFNγ, IL‐6,IL‐1β, and IL‐17a and upregulating anti‐inflammatory cytokine IL‐10	Eichenberger *et al*. (2018*a*)
miR‐21	Human colonocytes and mouse colonic crypts	Promotes cell proliferation and migration in human colonic epithelial cells and colonic crypts in wild‐type mice	Bakirtzi *et al*. (2019)
NEAT1	Mouse intestinal mucosa and serum	Involved in IBD pathogenesis hence a possible biomarker Downregulation of NEAT1 suppresses inflammatory response by modulating intestinal epithelial barrier and *via* exosome‐mediated polarization of macrophages in IBD	Liu *et al*. (2018*a*)
mdo‐miR7267‐3p	Ginger	Shapes gut microbiota Improves barrier function Ameliorates colitis *via* IL‐22‐dependent mechanisms	Teng *et al*. (2018)
miR‐4334, miR‐219 and miR‐338	Porcine milk	Prevents LPS‐induced intestinal inflammation, apoptosis and damage *via* inhibiting TLR4/NF‐κB and p53 pathways	Xie *et al*. (2019)
miR‐223	Mice colonic epithelial cells	Modulates communication between IL‐23 signal pathway and claudin‐8 in IBD development Modulates intestinal barrier integrity	H. Wang *et al*. (2016)
miR‐34c and PlncRNA1	Intestinal epithelial cell line Caco‐2	Modulates ZO‐1, MAZ and occludin expression Modulates intestinal barrier integrity	Chen *et al*. (2017)
mdo‐miR7267‐3p	Ginger	Shapes gut microbiome by gene targeting in *Lactobacillus rhamnosus* Increases expression of indole‐3‐carboxaldehyde (I3A) and IL‐22 Improves intestinal barrier function and ameliorates colitis	Teng *et al*. (2018)
GELNs lipids	Grapes	Induces Lgr5^+^ stem cells Enhances *in vivo* targeting of intestinal stem cells Remodels and protects intestinal tissue against DSS‐induced colitis	Ju *et al*. (2013)

DSS, dextran sulfate sodium; GELN, grape exosome‐like nanoparticles; IEC, intestinal epithelial cell; IFNγ, gamma interferon; IL, interleukin; LPS, lipopolysaccharide; MAZ, myc‐associated zinc finger protein; miR, microRNA; MSC, mesenchymal stem cell; NEAT1, nuclear paraspeckle assembly transcript 1; NF‐κB, nuclear factor kappa B; PlncRNA1, long non‐coding RNA PlncRNA‐1; TLR4, toll‐like receptor 4; ZO‐1, zona occludens‐1.

## V. IMMUNE CELL‐DERIVED EXOSOMES AND IBD

Exosomes derived from macrophages and monocytes provide an unparalleled opportunity to escape the control of mononuclear phagocytes to improve drug delivery to target sites and increase the therapeutic efficacy of drugs (Haney *et al*., 2015). Macrophages are polarized into M1 macrophages or M2 macrophages depending on the activation pathway. While classical activation leads to M1 macrophages which produce proinflammatory cytokines, the alternative leads to M2 macrophages which initiate anti‐inflammatory responses. The effects of exosomes derived from M2 macrophage subtypes M2a, M2b and M2c have been studied in experimental colitis in mice. The results show that M2b macrophage‐derived exosomes greatly mitigate the severity of the induced colitis by upregulating levels of regulatory T cells (Treg) and IL‐4, and downregulating levels of the colitis‐associated cytokines (IL‐6,IL‐1β, and IL‐17A) *via* the C‐C motif chemokine ligand 1/8 (CCL1/CCR8) axis (Yang *et al*., 2019). Wang *et al*. (2016*b*) conducted a study to investigate the influence of exosomes obtained from granulocytic myeloid‐derived suppressor cells on DSS‐induced murine colitis. Mice treated with the exosomes exhibited higher resistance to colitis and decreased inflammatory cell infiltration damage *via* the inhibition of T helper type 1 (Th1) cell proliferation and promotion of Treg expansion (Wang *et al*., 2016*b*).

The influence of exosomes derived from DCs treated with IL‐10 on colitis is also documented. It was shown that treatment with the exosome attenuated all the analysed macroscopic, clinical and histopathologic variables associated with colitis. Colonic tissue mRNA expression levels of IFN‐γ, TNF‐α, and IL‐2 were significantly reduced while IL‐10 mRNA and Treg expression levels increased (Yang *et al*., 2010). The mechanism by which these forkhead box P3 (Foxp3)‐positive Treg cells prevent diseases like IBD was investigated by Okoye *et al*. (2014) who showed that Treg utilizes a mechanism of non‐cell‐autonomous gene silencing *via* the mediation of miRNA‐containing exosomes to inhibit T‐cell‐modulated diseases. One specific miRNA implicated was let‐7d (Okoye *et al*., 2014).

## VI. STEM CELL‐DERIVED EXOSOMES AND IBD

Stem cell‐derived exosomes are increasingly implicated as regulators of several stem cell‐associated therapeutic effects. These exosomes are believed to elicit their therapeutic activities by delivering their cargo consisting of potentially therapeutic proteins and RNAs to recipient cells. One mechanism through which MSC‐derived exosomes perform their biological function of dampening inflammation is *via* ubiquitination (Wu *et al*., 2018). Exosomes obtained from MSCs of human umbilical cord significantly attenuate IBD by upregulating levels of IL10 and IFN‐γ inducible protein‐10 (IP10), while downregulating IL‐1β, TNF‐α, IL‐6,ubiquitin‐conjugating enzymes (E2M), NEDD8‐activating enzyme E1 (NAe1), and ubiquitin like modifier activating enzyme 3 (Uba3). Further analysis by western blot showed decreased expression levels of proteins such as K48, K63 and FK2 (indicating regulation of ubiquitin modification) in exosome‐treated IBD mice compared with controls (Wu *et al*., 2018). Treatment with MSC‐derived exosomes downregulates inflammatory responses, maintains intestinal mucosa barrier integrity and polarizes macrophages to the M2b phenotype, without favouring intestinal fibrosis (Liu *et al*., 2019).

It is known that neutrophil recruitment and activation within the intestinal mucosa contributes to IBD pathogenesis. Through their myeloperoxidase (MPO) activity, neutrophils migrate into the lamina propria of DSS‐induced colitis causing oxidative‐induced tissue damage and other detrimental effects (Segal, 2018; Zhou *et al*., 2018*a*). MSC‐derived exosomes reduce colonic MPO activities, indicating inhibited neutrophil invasion (Liu *et al*., 2019). Several other studies have indicated promising therapeutic effects of stem cell‐derived exosomes in IBD, including inhibition of T‐cell proliferation and differentiation, and promoting the apoptosis of activated T cells (Mokarizadeh *et al*., 2012; Baghaei *et al*., 2019). Bone marrow MSC‐derived exosomes also protect against IBD by reducing mRNA and protein levels of nuclear factor kappa B (NF‐κB), p65, iNOS, COX2, TNF‐α and IL‐1β but increasing levels of IL‐10. Furthermore, oxidation‐inducing factors like MPO and malondialdehyde (MDA) were downregulated while antioxidant factors like glutathione (GSH) and superoxide dismutase (SOD) were increased (Yang *et al*., 2015).

Similarly, exosomes effectively target colon tissues of IBD mice by 12 h post injection, and significantly attenuate the severity of IBD. Expression levels of IL‐10 increase whilst that of IL‐1β, IL‐6,IL‐7,TNF‐α, and iNOS genes decrease in both colon tissues and spleens of mice. Further analysis indicated that exosomes modulate this anti‐inflammatory effect partially through the regulation of IL‐7 expression in macrophages (Mao *et al*., 2017*b*). MSC‐derived exosomes have even been applied to experimental necrotizing enterocolitis; a medical condition in which a portion of the bowel dies. Rager *et al*. (2016) observed that treatment with either bone marrow derived‐MSCs or their extracted exosomes significantly decreased the incidence of necrotizing enterocolitis and intestinal permeability. Additionally, MSC‐derived exosomes profoundly enhanced wound healing in IEC‐6 cells (Rager *et al*., 2016).

## VII. FOOD‐DERIVED EXOSOMES (NANOVESICLES) AND IBD

Exosomes isolated from food sources elicit strong functional, developmental, and immunomodulatory effects on IECs. The nucleotide‐binding domain and leucine‐rich repeat‐containing family, pyrin domain‐containing 3 (NLRP3) inflammasome is a vital modulator of the innate immune response, and its abnormal stimulation is linked with the pathogenesis of several diseases including IBD. Its downstream effector proteins such as IL‐1β and caspase‐1 are known to exhibit protective or detrimental functions in mucosal immunity (Zhen & Zhang, 2019). In a recent study (Chen, Zhou, & Yu, 2019*a*), ginger rhizome‐derived exosome‐like nanoparticles strongly inhibited NLRP3 inflammasome activation. In addition, treatment with the vesicles hindered pathways downstream of inflammasome activation including secretion of IL‐1β and IL‐18, auto‐cleavage of caspase‐1 and pyroptotic cell death, consequently preventing the assembly of the NLRP3 inflammasome (Chen *et al*., 2019*a*). In another study, grape‐derived exosome‐like nanoparticles led to effects on intestinal tissue remodelling and protection (Ju *et al*., 2013). The lipid component of the vesicle typically aided in the induction or proliferation of Lgr5+ stem cells and *in vivo* targeting of intestinal stem cells. The treatment also accelerated organoid structure formation, triggered the activation of the Wnt/β‐catenin pathway and enhanced expression of stem cell growth‐related genes (Ju *et al*., 2013). Thus, these vesicles not only participate in the modulation of intestinal tissue renewal activities, but also in remodelling responses to pathological triggers.

The ability of exosome‐like nanoparticles to home effectively to the inflammatory microenvironment is controlled by C‐X‐C motif chemokine receptor 1 (CXCR1), CXCR2 and lymphocyte function‐associated antigen‐1(LFA‐1) (Wang *et al*., 2015*a*). Deng *et al*. (2017) documented the influence of broccoli‐derived nanoparticles in inhibiting induced colitis in mice. These exosome‐like particles mediate the activation of AMP‐activated protein kinase (AMPK) in DCs, which not only prevent DC activation but also induce DC tolerance, resulting in inhibited DSS‐induced colitis in all mouse models used (Deng *et al*., 2017). Milk‐derived exosomes obtained from humans (Martin *et al*., 2018) and pigs (Xie *et al*., 2019) have also been shown to attenuate cell death and inflammation in IECs by inhibiting TLR4/NF‐κB and p53 pathways in the intestinal epithelium.

## VIII. THE DOUBLE ROLE OF IEC‐DERIVED EXOSOMES IN IBD

Depending on the source, functional composition, micro environmental factors and other immunological factors, exosomes exhibit either anti‐inflammatory or pro‐inflammatory effects leading to attenuation or progression of diseases including IBD. Under normal physiological conditions, exosomes expressed by IECs help to maintain intestinal homeostasis and functions. They participate in modulating resident immune cells, the gut microbiota, IEC proliferation and barrier integrity. Normal IECs secrete EVs that exhibit transforming growth factor beta‐1 (TGF‐β1)‐dependent immunosuppressive activity within the mucosal microenvironment (Jiang *et al*., 2016). Administration of these vesicles attenuates IBD severity *via* induction of both Treg and immunosuppressive DCs. They are also found to be epithelial cell adhesion molecule (EpCAM) dependent and show upregulated levels of TGF‐β1 during IBD development in an ERK‐dependent manner (Jiang *et al*., 2016). This demonstrates the involvement of IECs in the maintenance of intestinal tract immune balance and intestinal homeostasis.

By contrast, exosomes secreted from sites of intestinal inflammation in IBD are known to promote proinflammatory factors. It is known that intestinal luminal aspirate‐derived exosomes from IBD patients contain markedly higher mRNA and protein levels of IL‐6,IL‐8, and TNF‐α than those of healthy controls. These EVs are taken up by colonic epithelial cells, resulting in upregulation of IL‐8 protein and subsequent induction of macrophage migration by the epithelial cells (Mitsuhashi *et al*., 2016). Adherent and invasive *E. coli* is known to be present at high levels in the intestinal mucosa of CD patients and damages intestinal epithelial barrier integrity by regulating and disorganizing cell junction proteins to increase permeability. Exosomes expressed from these infected IECs participate in activation of the host innate immune response as well as enhancing intracellular replication of these pathogens. Activated macrophages further induce the secretion of exosomes that help to promote the proinflammatory response (Carrière *et al*., 2016). Chen *et al*. (2019*b*) report that exosomes derived from a dysbiotic gut microenvironment (due to triggered intestinal mucosal injury) can cause hepatic steatosis in a mouse model. These exosomes expressed by the gut had significantly high levels of high‐mobility group box‐1 (HMGB1) and were transported *via* the gut–liver axis to establish the hepatic steatosis (Chen *et al*., 2019*b*). On the basis of these reported dual activities of IEC‐derived exosomes, there is a need to investigate the factors and mechanisms that induce the proinflammatory‐associated exosomes. Treatment targets against such factors or mechanisms would be a novel approach in the treatment of IBD and other intestinal inflammatory diseases.

## IX. FUNCTIONAL MODIFICATION OF EXOSOMES

**Fig 5:**
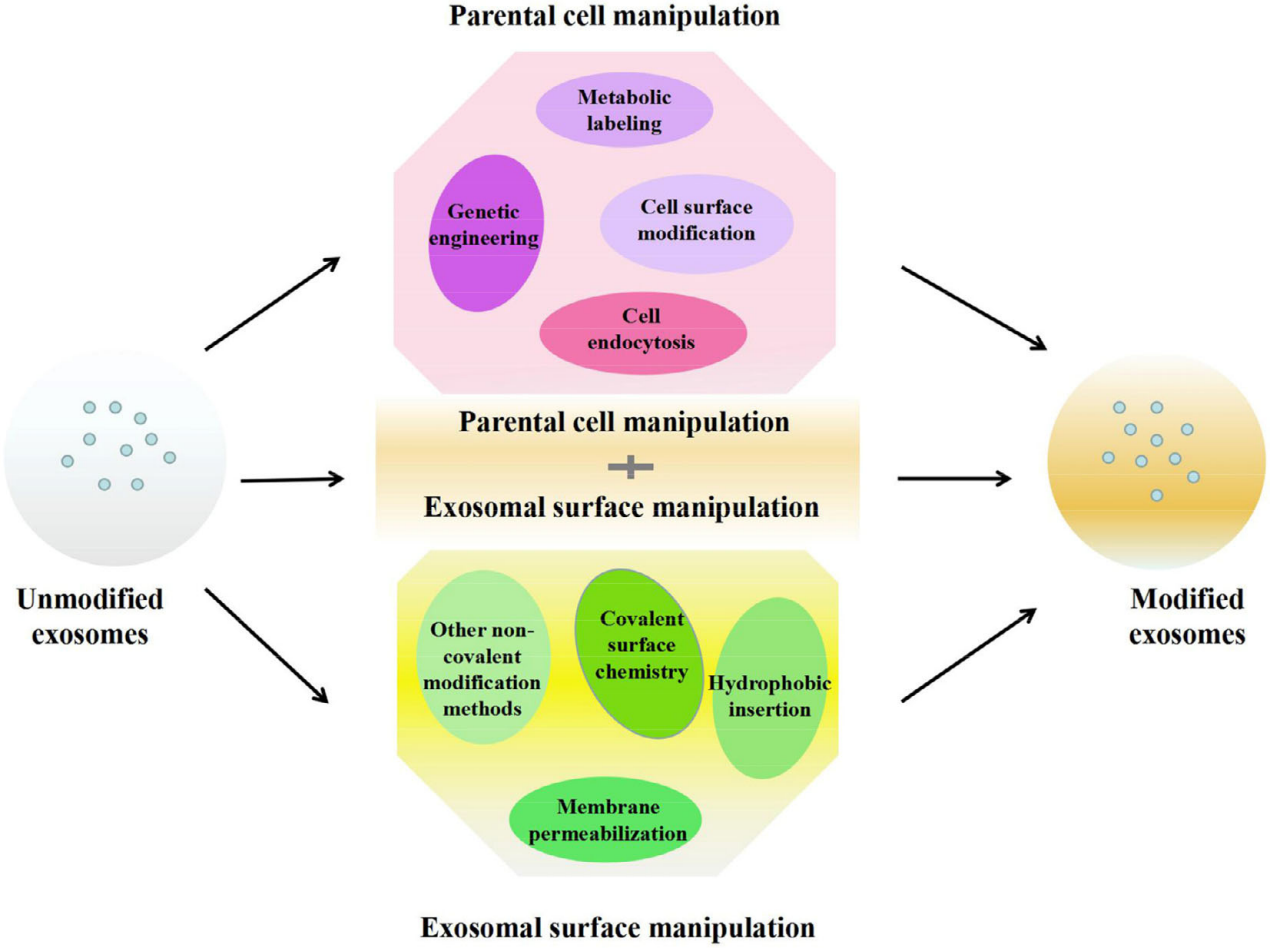
Functional modification of exosomes for use in therapeutic applications. The manipulation of exosomes can be classified into three broad groups: modification of cells before they secrete the exosomes; direct modification of exosomes; and a combination of cellular and exosomal manipulation techniques. The final product could elicit enhanced target specificity and improved therapeutic efficacy.

With their intrinsic biodegradability and molecular payload, modification of exosomes creates exciting new ways to modulate cellular responses and develop novel nano‐delivery systems in precision therapeutics (Zhu *et al*., 2018). Such engineered nanovesicles could enhance immunomodulation towards tissue regeneration and repair within the IBD microenvironment. They could also be utilized in the diagnosis and imaging of disease. The modification of exosomes in nanomedicine can be classified under three methods: cell cargo loading modification (parent cell manipulation); exosome membrane surface modification (direct EV functionalization); and integrated cell cargo loading and exosome membrane surface modification (Fig. 5).

### (1) Current methodological approaches

Cargo loading modification (parent cell manipulation), genetic, physical or chemical manipulation techniques are employed to load proteins, RNAs, drugs and other organic or inorganic molecules into the vesicles *via* the manipulation of parental cells. Armstrong, Holme, & Stevens (2017) review these modification strategies and include specific manipulation methods such as genetic engineering, which promotes gene expression and regulates transcription within recipient cells, and metabolic labelling, in which desired molecules or functional groups in the form of metabolite analogues are added to the cell's biosynthesis. The packaging of exogenous materials into vesicles can be achieved through liposome or micelle fusion with cytoplasmic membranes. Alternatively, these molecules can be incorporated into EVs by hijacking the normal process of packaging endocytosed materials into EVs (Armstrong *et al*., 2017).

EV surface modification entails the decoration of vesicular surfaces with bioactive molecular particles *via* covalent and non‐covalent manipulation methods as well as genetic methods and hydrophobic insertion. To this effect, EVs have been engineered to load molecules (including drugs) into their interior. Similarly, hydrophobic insertion has been used to insert lipophilic and amphiphilic molecules into vesicular membranes. Moreover, it is possible to modify EV membranes to become surfaces for chemical reactions. A typical example is click chemistry reactions, usually used in bio‐conjugation, allowing the joining of substrates of choice with specific biomolecules on the membrane. In one study, an active alkynyl group was incorporated into exosomes by carbodiimide coupling, then attached to an azide‐labelled fluorophore545 on the exosomes by click chemistry reaction (Smyth *et al*., 2014).

Currently, several research teams use an integrated cargo loading and membrane surface modification strategy to manipulate EVs to achieve optimal effects. Sato *et al*. (2016) reported that exosomes embedded with specific membrane proteins isolated from genetically modified cells could successfully fuse with several liposomes, confirming that genetic modification (parental cell manipulation) techniques can be integrated or combined with membrane manipulation methods.

### (2) Application of the modified exosome and exosome‐like vesicles in IBD

Based on the important roles of IL‐10 in the development of normal mucosal immunity, researchers have investigated the potential for exosomes derived from DCs treated with IL‐10 to suppress TNBS‐induced colitis. The modified exosomes were administered intraperitoneally and the results assessed after 3 days. There was significant reduction in all clinical, histopathological and macroscopic variables associated with the induced colitis. This was noted to be linked with downregulated expression of mRNAs of IL‐2,TNF‐α and IFN‐γ within the colonic tissues. This treatment resulted in significantly upregulated expression of IL‐10 mRNA and Treg in colon tissues and colonic lamina propria, respectively (Yang *et al*., 2010). Similarly, intraperitoneal injection of exosomes derived from DCs treated with *Schistosoma japonicum* egg antigen significantly attenuated the severity of induced IBD in mice more effectively than untreated DC exosomes (Wang *et al*., 2017). Yu *et al*. (2013) also genetically modified DCs to overexpress TGF‐β1. Exosomes derived from these cells exhibited a strong immunosuppressive capacity by impairing Th1 and IL‐17 responses, while promoting IL‐10 responses (Yu *et al*., 2013).

Lymphangiogenesis plays an important role in the pathogenesis of IBD, and vascular endothelial growth factor‐C(VEGF‐C) is a strong factor promoting lymphangiogenesis. Adipose‐derived stem cell exosomes preconditioned with VEGF‐C trigger significantly higher levels of miR‐132 in exosomes. Treatment of lymphatic endothelial cells (LECs) with the VEGF‐C‐modified exosomes led to enhanced LEC migration, proliferation and increased tube formation compared with unmodified exosomes (Wang *et al*., 2018). The same study demonstrated that miR‐132 promotes lymphangiogenic responses *via* direct targeting of mothers against decapentaplegic homolog 7 protein (Smad‐7) and regulation of TGF‐β/Smad signalling (Wang *et al*., 2018). Further studies on the mechanisms and molecules involved in this process could lead to novel approaches to IBD treatment.

Modified exosome‐like vesicles from food have also been applied in IBD treatment. Modified grapefruit‐derived nanovectors sufficiently targetted inflammatory sites with enhanced chemotherapeutic effects as indicated by inhibition of inflammatory effects in DSS‐induced mouse colitis and prevention of tumour growth (Wang *et al*., 2015*a*). Engineered embelin lipid nanospheres show positive treatment outcomes in acetic acid‐induced UC (Badamaranahalli *et al*., 2015). In this case, soya bean oil/virgin coconut oil was used as a liquid lipid carrier and soya/egg lecithin as a stabilizer to engineer vesicles *via* hot homogenization followed by ultrasonication. In addition to the confirmed improved histopathology of colonic tissues, expression levels of MPO, lipid peroxidation (LPO) and lactate dehydrogenase (LDH) were decreased while that of GSH increased, implying a better treatment outcome (Badamaranahalli *et al*., 2015). Zhang *et al*. (2017) developed a novel small interfering RNA (siRNA) delivery system which was used to load siRNA‐CD98 into ginger‐derived nanoparticles. Oral administration of the engineered vesicles efficiently and specifically targeted colon tissues and reduced expression levels of CD98 (Zhang *et al*., 2017).

## X. EXOSOMES AS NANOCARRIERS

Over recent years, nanobased drug‐delivery systems have gained considerable importance. Different techniques have been employed to enhance the therapeutic efficacy of biomolecular and chemical drugs. In addition to delivering therapeutic proteins, RNAs and other molecules, exosomes have also been engineered to deliver imaging particles to sites of interest. They have been identified as crucial mediators of distant intercellular communication and participate in a diverse range of biological activities and processes. They are noted to have an ideal native structure and characteristics that make their use as nanocarriers in clinical and experimental settings highly promising. Among these special characteristics are their small size making it easy to penetrate into deep tissues, pliable cytoskeleton, high probability of escaping degradation or immune system clearance, and their resemblance to cell membranes (Hood, 2016). The two major mechanisms by which therapeutic substances are incorporated into exosomes are passive and active encapsulation, which both result in different drug‐loading stabilities and efficiencies.

### (1) Passive encapsulation

In this method, drugs are incubated with either parental cells or extracted exosomes. The drugs move *via* a concentration gradient into the extracted exosomes or into the parental cells and trigger the cells to secrete exosomes loaded with the treated drugs. For example, when MSCs were treated with Paclitaxel, the extracted exosomes were found to contain significant amounts of the drug and showed greater efficacy than untreated cells (Pascucci *et al*., 2014). Sun *et al*. (2010) reported that the passive encapsulation of curcumin into exosomes heightened their anti‐inflammatory effects. They further validated that employing exosomes as a delivery vehicle for the drug made it highly stable, more concentrated in plasma and more target‐specific to inflammatory cells. In the delivery of anti‐inflammatory drugs like curcumin into the IBD microenvironment, factors such as pH are also important to consider (Beloqui *et al*., 2014).

### (2) Active encapsulation

Active encapsulation relies on drug molecules (the active agent) crossing the membrane of the exosome into the core and becoming entrapped. To achieve this, several methods have been used to compromise membrane integrity to allow diffusion without affecting the inherent structure and functions of the exosome. Methods such as extrusion and sonication temporarily disrupt the membrane structure and increase porosity, allowing the drug molecules to enter the exosome (Liao *et al*., 2019). Other methods include electroporation, freeze and thaw cycles and the use of membrane‐permeable substances. Electroporation utilizes an electrical field to create small pores in the phospholipid bilayer, allowing transient diffusion of the drug followed by recovery of the exosome membrane after loading. The use of surfactants like saponin both increase the membrane permeability of exosomes and also preserve the activities of the loaded drug (Haney *et al*., 2015). Freezing at −80°C followed by thawing has also been used successfully to load particles into exosomes as has engineering hybrid exosomes by membrane fusion of liposomes and exosomes (Sato *et al*., 2016).

### (3) Application of exosomes as nanocarriers in IBD

The use of exosomes in nanotechnology is increasingly becoming a promising technique for the treatment of IBD. Although the inflamed colonic environment presents challenges such as a disrupted epithelium, a thick mucus layer, and altered colonic transit time, it is generally reported that nano‐sized drug‐delivery systems are able to overcome such barriers, leading to increased accumulation within the inflamed tissue. This ultimately leads to increased quantities of the drug in the colon with corresponding increases in efficacy and a reduced likelihood of systemic side effects (Beloqui, Coco, & Préat, 2016). Endogenous annexin A1 (ANXA1) is produced as a component of EVs obtained from IECs. This protein is known to regulate intestinal mucosal injuries, inflammation and repair (Babbin *et al*., 2008). In nanocarrier technology, local administration of exogenous ANXA1 mimetic peptide, encapsulated within targeted polymeric nanoparticles accelerates healing of murine colonic wounds. In same study, a single systemic administration speeds up recovery after an experimentally induced colitis (Leoni *et al*., 2015). Oral administration of PLGA/PLA–PEG‐FA nanoparticles loaded with the ginger active compound 6‐shogaol (NPs‐PEG‐FA/6‐shogaol) in a colitis model significantly mitigates colitis symptoms and accelerates colitis wound repair by modulating the anti‐inflammatory factors nuclear factor erythroid 2‐related factor 2 (Nrf‐2) and heme oxygenase‐1 (HO‐1), and the inflammatory cytokines TNF‐α, IL‐1β, IL‐6 and iNOS. NPs‐PEG‐FA was also shown to be highly biocompatible with efficient receptor‐mediated uptake by activated Raw 264.7 macrophages and colon‐26 cells (Zhang *et al*., 2018a).

## XI. EXOSOMES AS BIOMARKERS OF IBD

The presence of certain exosomes or their components has been implicated in the establishment of several diseases including IBD. Studies in this field continue to show promising results. Some such studies have identified exosomes in the placenta as early biomarkers of preeclampsia (Salomon *et al*., 2017), exosomal microRNAs and circulating exosomes as biomarkers of gastrointestinal cancer (Nedaeinia *et al*., 2017), and circulating exosomes as biomarkers of cardiovascular diseases (Parizadeh *et al*., 2019). Other conditions in which exosomes have been identified as potential biomarkers include diabetes type 1 (Garcia‐Contreras *et al*., 2017), breast cancer (Jia *et al*., 2017) and prostate cancer (Duijvesz *et al*., 2011) among others.

A number of biomarkers are known to be relevant to IBD, however, none have yet been identified as a single biomarker for its diagnosis or that can differentiate UC patients from CD patients with sufficient sensitivity and specificity. Significant among these biomarkers are anti‐*Saccharomyces cerevisiae* antibodies (ASCA), perinuclear anti‐neutrophil cytoplasmic antibodies (pANCA), C‐reactive protein (CRP), lactoferrin, and calprotectin (Bennike, 2014). According to Zheng *et al*. (2017), salivary exosomal proteasome subunit alpha type 7 (PSMA7) shows promise as a biomarker for IBD when exosomes from both IBD patients and healthy individuals are compared, and expression levels of this exosomal protein are higher in CD patients than UC patients. Leoni *et al*. (2015) report that in comparison with healthy controls, IBD patients express high levels of endogenous ANXA1‐containing EVs. Given that ANXA1‐containing EVs increase in circulation in response to the inflammatory process, Leoni *et al*. (2015) noted that it could potentially serve as a biomarker of intestinal mucosal inflammation. In another study, EVs obtained from intestinal luminal aspirates of an IBD patient were estimated to have a mean concentration of 4.3 × 10 particles/ml and a mean diameter of 146 nm. Their stability in luminal samples as well as their protein and mRNA content identify them as a potential faecal biomarker that reflects mucosal inflammation (Mitsuhashi *et al*., 2016). Liu *et al*. (2018*a*) suggest a potential application of the exosomal RNA NEAT1 as a biomarker for the diagnosis and subsequent treatment of IBD, however detailed examination of the mechanisms underlying the participation of NEAT1 in the pathogenesis of IBD are still needed. The ability to identify a highly specific and sensitive biomarker for IBD would not only promote early‐stage and accurate diagnosis, but could also improve treatment, prognosis and potentially lead to the discovery of biological pathways upon which novel therapies could be developed. There is much to be gained from further investigations of these markers and their associated molecules and pathways.

## XII. DISCUSSION

While stem cell‐based therapy has achieved significant success in its applications in regenerative medicine, challenges remain and only a few cellular therapies have obtained approval for sale worldwide despite huge investment (Cuende *et al*., 2018). Adverse outcomes have been documented in the use of stem cells in clinical trials, including the development of tumours in a kidney failure patient, and blindness in a macular degeneration patient (Kuriyan *et al*., 2017). These, among other factors elaborated in a recent review (Ocansey *et al*., 2020), have led to a new focus on exosomes as packages of beneficial biomolecules released by stem cells and other cells. Exosomes act naturally as intercellular messengers that promote communication between cells. Their release by healthy cells following activation, or constitutively, results in important immunomodulatory functions. These vesicles are essential for healthy physiology, however under certain pathological conditions, they can potentiate cellular stress and damage (Isola & Chen, 2016).

The use of exosomes as an alternative to stem cell therapies is likely to be simpler, safer and lower cost; they are more easily stored and transported and their therapeutic application avoids many of the problems associated with the administration of parental stem cells. Additional benefits include the fact that they pose less risk of triggering adverse immune responses, they are non‐oncogenic, have high stability, cannot replicate (hence are incapable of transforming into malignant cells or other harmful cell types), and cannot be infected with viruses. They also possess high tissue‐specific homing capacity and a low vascular‐obstructive propensity. In relation to IBD, exosomes are known to regulate immune cells and cytokines within the inflammatory microenvironment. This modulation results in dampened inflammation, restored intestinal barrier integrity and restored gut microbiome composition and diversity. Systemic injection of exosomes at the onset of colitis and during chronic colitis decreases disease severity. Exosome infusion also inhibits the recurrence of DSS‐induced IBD following subsequent DSS administration (Liu *et al*., 2019). The anti‐inflammatory activities of exosomes include the polarization of macrophages into the M2 phenotype, which can result in a severe and distressing complication of IBD called intestinal fibrosis (Lawrance *et al*., 2015). The existing data do not implicate the therapeutic application of exosomes in the formation of intestinal fibrosis (Liu *et al*., 2019), although this requires further investigation.

The human intestinal mucosal surface represents the primary defence against pathogens, and modulates the immune response *via* resident immunological factors as well as IEC functions. IECs act as sensors of luminal stimuli, interacting with the gut microbiome and immune cells through signalling transduction pathways, thus representing the first barrier that food, pathogens and chemicals that trigger IBD encounter. Exosomes crucially participate in this IEC–microbiome–immune system interaction to maintain mucosal homeostasis. Alterations to intestinal exosome proteomics alone can lead to abnormal host–gut microbiota interactions causing dysbiosis (Zhang *et al*., 2018*b*). Several studies have demonstrated the regulatory effects of exosomes on restoring gut microbiota composition and diversity, as well as modulating gene transcription and growth of gut bacteria. In the host immune system, exosomes generally induce Treg, regulatory DCs and M2 phenotype macrophages, resulting in immunosuppressive effects within the IBD microenvironment. These vesicles directly modulate IEC development, proliferation, and differentiation, as well as tight junction molecules. These powerful modulatory effects of exosomes within the IBD microenvironment make them ideal candidates for treating and preventing the recurrence of IBD.

Notwithstanding, some studies have demonstrated pro‐inflammatory effects of exosomes (mainly from infected cells or IBD patients) on intestinal tissues and immune cells like macrophages. This shows that both anti‐inflammatory‐mediating exosomes and pro‐inflammatory‐mediating exosomes are found within the IBD microenvironment. Pro‐inflammatory‐mediating exosomes and their cargos seem to be enriched during active IBD, hence the continued inflammation. The balance between these vesicles in the mucosal microenvironment likely plays an important role in inducing, maintaining and regulating the required functions of intestinal tissues (Kubiritova, Radvanszky, & Gardlik, 2019). Targeting and subsequently inhibiting such exosomal constituents and mechanisms or other microenvironmental factors involved in the excessive pro‐inflammatory effects of IBD would be a novel approach in both its treatment and diagnosis. Factors associated with increased expression of anti‐inflammatory‐mediating exosomes in intestinal tissues, resident immune cells and the gut microbiota should also be investigated.

Clinical application of nanobased drug‐delivery systems is faced with two distinct problems: rapid clearance by the mononuclear phagocyte system (MPS) or the reticuloendothelial system (RES), and cytotoxicity. Although PEGylation could increase the circulation period of such nanoparticles, it could potentially inhibit interactions between the drug‐delivery system and target cells, hence reducing the drug's bio‐distribution in diseased tissues (Suk *et al*., 2016). In light of this, the use of naturally equipped (endogenous) nanocarriers like exosomes as compared to synthetic nanoformulations shows promise in improving drug delivery, consequently leading to higher therapeutic efficacy due to their natural bio‐compatibility *in vivo* (Batrakova & Kim, 2015; Peng & Mu, 2016). Several natural nanoformulations have been applied in the treatment of IBD including curcumin, ginger‐derived nanoparticles, silymarin, quercetin, embelin, grape exosome‐like nanoparticles and natural polysaccharides like pectin, cellulose, chitosan and dextran. They exhibit high antioxidant and anti‐inflammatory properties which enable them to modulate various inflammatory mediators efficiently, including TNF‐α, IL‐6,IL‐1β, IL‐10, prostaglandin E2 (PGE‐2), iNOS, and COX‐2 (Taghipour *et al*., 2018). The application of both natural and synthetic nanoparticles and microparticles including exosomes has demonstrated enhanced bioavailability, specificity, stability and biodistribution (Laroui *et al*., 2010). This method of drug delivery thus has high prospects of prolonged and stable remission in patients and reduced overall drug administration. Clinical trials will allow us to explore the use of nanobased drug‐delivery products for the treatment of IBD.

The healthy subject and IBD patients release exosomes containing varying concentrations of proteins, RNAs and other constituents into circulation which could be measured as biomarkers. The abnormal expression of such biomarkers within the serum and tissues of IBD patients would indicate the onset of a molecular and genetic imbalance originating from either reduced regulatory molecules or enhanced pro‐inflammatory mediators. Although few of these exosomal biomarkers are documented to date, there is great potential for the use of these exosomal components and their targets as novel diagnostic and therapeutic (by utilizing inhibitors and mimics) tools for IBD.

## XIII. FUTURE PERSPECTIVES

The highly potent modulatory effects of exosomes within the IBD microenvironment are clearly evident and well documented. Notwithstanding, future studies should aim to provide a better understanding of the mechanisms and factors involved. There are no clinical trials yet published on the use of this therapeutic tool in IBD. We also still require studies on the best exosome preparation method, administration route and dosage, among other factors. While stem cells and other cells produce large quantities of exosomes naturally, effective separation and purification of these continues to be difficult. There is a need to focus attention on the optimal isolation and purification procedures to allow the production of a well‐defined set of pharmaceutical‐grade exosome products as a next‐generation cell‐free therapy in regenerative medicine. The high biocompatibility of exosomes will provide greater opportunities for clinicians and medical nutritionists to develop safe and targeted therapies for the treatment and management of various conditions. Combined therapy of exosomes (or their cargos) and other existing IBD therapies should also be explored in our search for successful clinical applications.

## XIV. CONCLUSIONS

1. Chronic inflammation of the bowel is characterized by immune dysregulation, dysbiosis, and continuous destruction of IECs leading to compromised barrier integrity.
2. IBD therapies seek to regulate microenvironmental factors associated with the inflammation to restore balance between inflammatory and anti‐inflammatory elements.
3. As an emerging therapeutic option for IBD, exosomes efficiently modulate immune system cells, the gut microbiota and IEC barrier functions to attenuate IBD.
4. Exosomes share a common basic structure mainly consisting of proteins, RNAs and lipids. These constituents are responsible for the specific effects elicited by exosomes including the possibility that they may have a clinical application as biomarkers of IBD.
5. These EVs can be manipulated chemically or biologically to broaden, change and enhance their therapeutic capability, including their use as nanocarriers or teranostic platforms with high target‐specificity.
6. Given the well‐documented and promising outcomes of exosome‐induced modulation in IBD animal models, the stage is set for clinical trials. Future work should also continue to explore the mechanisms by which exosomes can be modulated in the hope of identifying novel therapeutic and diagnostic tools in the treatment of IBD.
